# Real-World Application of Non-Destructive Pavement Health Monitoring Sensors

**DOI:** 10.3390/s26030899

**Published:** 2026-01-29

**Authors:** Alessandro Di Graziano, Salvatore Cafiso, Filippo Praticò, Enea Sogno, Andrea Fontana

**Affiliations:** 1 Department of Civil Engineering and Architecture, University of Catania, 95125 Catania, Italy; dcafiso@unict.it; 2 DIIES Department, University Mediterranea of Reggio Calabria, 89124 Reggio Calabria, Italy; filippo.pratico@unirc.it; 3 SINA S.p.A., 20135 Milano, Italy; 4 ITER S.r.l., a Spin-off of the University of Catania, 95125 Catania, Italy

**Keywords:** pavement monitoring, sensors, acoustic signature, structural health monitoring

## Abstract

Monitoring road pavement conditions is a fundamental activity in road network management. A well-structured pavement management system (PMS) is based on the continuous collection of data on pavement conditions throughout the road’s life cycle. In recent years, the integration of sensor technologies into road pavement for condition monitoring has attracted increasing attention. The collection of such data allows the construction of models that describe pavement deterioration as a function of traffic loads. This study presents an innovative solution (NDSPHM) for monitoring the structural condition of road pavements, which involves using acoustic sensors (microphones) to acquire the signature generated by passing vehicles, which propagates through the pavement structure. In more detail, this work focuses on the processing methodology applied to data collected on a highway under real traffic conditions.

## 1. Introduction

The quality of national and international road networks is of great economic and social value. As a result, the adequate maintenance of transportation systems is essential to preserve and enhance these benefits. The management of road pavements is generally carried out through a Pavement Management System (PMS) [1], which enables infrastructure agencies to define intervention strategies based on objective data and techno-economic evaluations, and to set the priorities and the scheduling of interventions over a multi-year planning scenario.

A good PMS is based on in-depth surveys to determine both the structural and functional condition of the pavement, performed periodically during its life.

Conventional methods involve destructive methods like core sampling combined with the road engineer’s/expert’s visual inspections of the road, as well as in situ (ND) tests, using devices like Falling Weight Deflectometer (FWD), Light Weight Deflectometer (LWD), and Ground Penetrating Radar (GPR). These methods, however, are expensive operations and often require the temporary closure of the road section under investigation. These limitations have prompted the development of Intelligent Transportation Systems (ITS) [2], which incorporate sensor-based technologies for continuous and automated pavement condition assessment [3]. Embedding these sensors directly into road structures offers a valuable tool for infrastructure managers seeking to enhance asset management strategies [4].

Over recent years, various monitoring solutions have been proposed, including the use of Smart Sensor Networks (SSNs) embedded within or mounted on the pavement surface. Embedded systems enable high-precision assessment of internal pavement conditions but must be installed during construction phases and often suffer from reduced lifespan due to harsh environmental conditions [5,6]. By contrast, non-embedded sensors operate non-destructively, typically measuring surface parameters and inferring internal conditions from these measurements [7,8]. Mobile monitoring platforms, capable of evaluating large road sections without interfering with traffic flow, offer averaged assessments but often entail high costs and complex data processing requirements [9,10]. Stationary systems provide more accurate, point-specific measurements but may disrupt traffic during their operation [11,12].

Regarding connectivity, wireless sensor systems enable wide area coverage without the need for physical cabling, however they require secure communication protocols to ensure data integrity and privacy [13,14]. Wired systems are less susceptible to power or connection failures but tend to be intrusive and unsuitable for long- data transmission [15,16]. From an energy perspective, self-powered systems are practical for short-term monitoring but are constrained by their limited energy autonomy, making them less effective for long-term deployment [6,17]. Furthermore, conventional data management approaches based on sampling, filtering, and manual interpretation do not support automated insight extraction from collected datasets [18,19]. In contrast, intelligent data processing techniques based on artificial intelligence can produce meaningful insights and enable secure and efficient data transmission. However, these methods require significant computational resources and face challenges related to the complexity and volume of sensor-generated data [20,21].

Recent advances in pavement structural health monitoring have highlighted the strong potential of fibre-optic sensing technologies, particularly Fiber Bragg Grating (FBG) sensors, for in situ and real-time measurement of pavement mechanical response. Several studies have shown that FBG-based systems, embedded within asphalt or cement-treated layers, can effectively detect traffic-induced strain variations and support the identification of axle passages and load-related characteristics. These high-resolution, point-based strain measurements can be calibrated and validated against traditional non-destructive methods, such as FWD testing, to support structural assessment and performance evaluation [22].

More recently, research has explored hybrid approaches that combine discrete fibre-optic sensors with distributed fibre-optic sensing (DFOS) techniques. In particular, systems based on optical frequency domain reflectometry (OFDR) enable quasi-continuous strain measurements along the fibre, providing detailed insight into strain distribution and damage evolution, while FBG sensors can supply high-frequency point measurements suitable for capturing dynamic and transient effects [23].

Despite their high sensitivity and measurement accuracy, fibre-optic solutions generally require invasive installation, as the sensors must be embedded within structural layers. Moreover, they often require robust temperature-compensation strategies and specialized interrogation units, aspects that in many practical contexts may hinder large-scale, network-level implementation for long-term pavement monitoring.

This study aims to introduce an efficient, low-cost, and non-invasive methodology for the passive monitoring of road infrastructure, with particular emphasis on the early detection of structural anomalies in the subsurface layers of pavements. In this context, “low cost” refers to the total long-term cost rather than the initial installation investment. Once installed, the NDSPHM system enables continuous traffic-induced monitoring, reducing the need for repeated NDT campaigns (e.g., FWD/HWD) and invasive interventions; moreover, its modular configuration may limit downtime and maintenance costs, making it potentially advantageous for large-scale road networks.

The proposed framework is built upon a series of prior investigations that have informed the development of both the data acquisition architecture and the associated analytical procedures. These foundational studies have validated the feasibility and utility of sensor-based approaches for road condition assessment (see, e.g., Fedele et al., 2019 [24]; Cafiso et al., 2020 [25]; Praticò et al., 2020 [21]; Fedele et al., 2020 [26]; Di Graziano et al., 2021 [27]).

More specifically, the proposed method focuses on the analysis of the pavement’s “structural signature” and its dynamic response to mechanical and acoustic stimuli occurring under ordinary traffic conditions. In particular, the spectral characteristics of an acoustic signal transmitted through an intact pavement differ from those observed in a damaged pavement. The methodology therefore exploits the vibro-acoustic signature of the road surface, recorded by acoustic sensors embedded within properly waterproofed road markers positioned on the pavement surface.

The development of the data acquisition and transmission system, from conceptual design to implementation, was carried out by ITER S.r.l., a spin-off of the University of Catania in collaboration with the Laboratory for Road, Railway, and Airport Materials Testing at the Mediterranean University of Reggio Calabria.

This article first presents an overview of the prototype designed for road pavement monitoring, followed by a detailed description of the data management methodology and the results obtained from a field test conducted on an active roadway. Finally, the study explores potential applications and future development prospects for the monitoring system.

## 2. System Architecture and Installation

**Figure 1:**
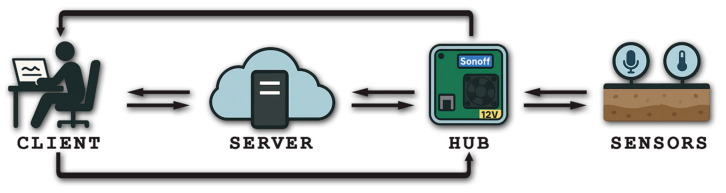
System.

The prototype for data acquisition and transmission (Non-Destructive Structural Pavement Health Monitoring, NDSPHM), developed in the laboratory and tested on a road open to traffic, comprises at least three components: a local unit, connected to a central unit (Hub), both capable of exchanging data with a dedicated server linked to one or more system users (clients) (Figure 1).

To ensure complete coverage of the infrastructure being monitored, it is necessary to install multiple units along its length to achieve complete mapping.

The central units receive and send data to/from the local units and to/from the server. Physically, they consist of a suitably modified case measuring 263 × 185 × 95 mm, equipped with a cooling system that ensures a stable temperature for proper component operation. Inside the case are the various electronic components, with the microprocessor relying on a Raspberry Pi 4 (RPi 4), a high-performance development board comparable to a minicomputer. Connected to this development board is an audio board, which enables analog-to-digital conversion and amplification of vibro-acoustic signals acquired from sensors installed in the pavement.

The central unit is powered at 12 V DC through a dedicated battery pack, which ensures continuous operation even in the absence of a direct connection to the electrical grid. The system’s autonomy represents a distinctive and essential feature for deployment in road environments, where mains power availability may be limited in certain situations. An integrated converter manages the conversion and regulation of the input voltage, ensuring proper power supply to the various electronic components operating at different voltage levels.

As part of the device development, a remote power-control system has also been implemented within an IoT framework using a wireless controller, allowing the power supply to be switched on and off remotely via a mobile application and enabling recovery operations without on-site intervention. Further enhancements to the power subsystem (e.g., integration of photovoltaic panels) are currently under evaluation to further increase autonomy.

The local units are roadside sensors that collect structural and environmental information, managed by the central units, which communicate wirelessly with the operator.

Specifically, the local units are equipped with acoustic sensors (microphones) to capture vibro-acoustic signals from the pavement. The surface temperature of the pavement layers is monitored using thermocouples installed at the pavement surface, one for each local unit.

**Figure 2:**
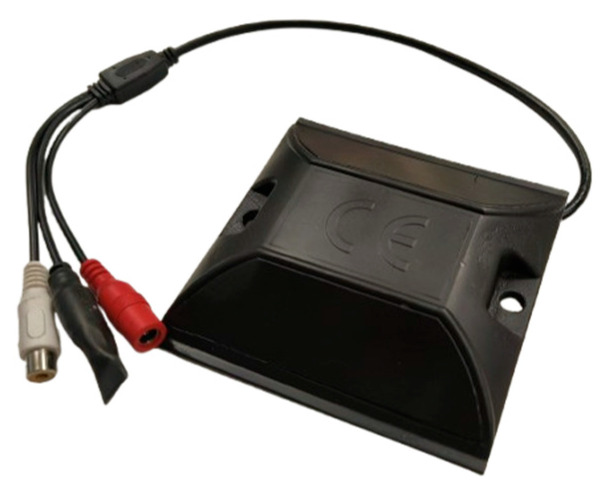
Sensor modified to be buried.

A surface installation configuration has been developed for the acoustic sensors, which involves embedding them within road markers (road studs), properly waterproofed, and positioned on the road pavement surface (Figure 2). Connection to the centralized unit is provided by routing the necessary cabling through buried corrugated tubing that extends from a buried junction box near the sensors.

Furthermore, to ensure the proper functioning of the entire system, high-reliability security protocols are employed for communication between the central unit, the local units, and the server.

**Figure 3:**
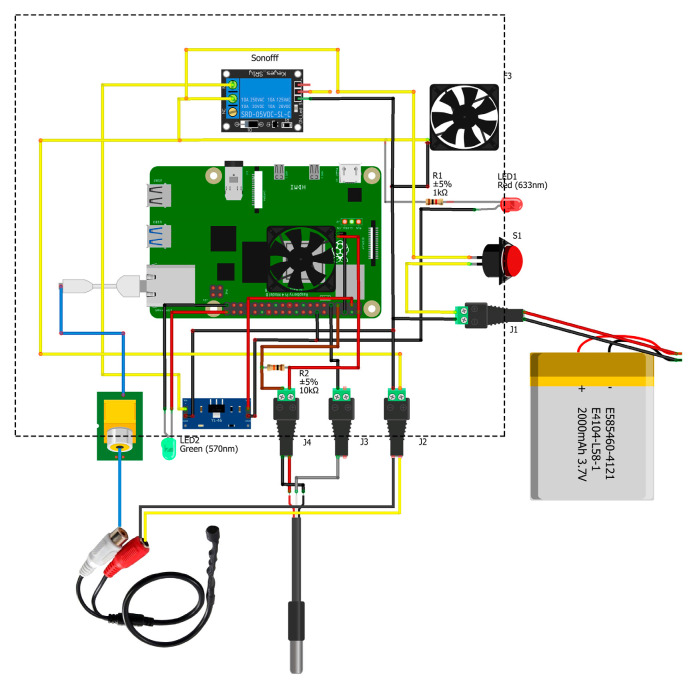
Hardware scheme.

Figure 3 provides an overview of the device architecture, specifically offering a high-level representation of the main components of the central unit.

## 3. Data Management

The processing of data collected from the buried sensors proves to be a complex and lengthy operation due to the vast quantity and variety of information generated, commonly referred to as “Big Data”.

This procedure was developed through an initial experimental phase involving manual selection and classification, and based on the results of various tests, it was later automated using a programming language (Python 3.13.1). This resulted in a solid foundation for analysing large volumes of data, supporting future developments, and enabling intelligent infrastructure monitoring.

The system, based on the responses of the monitored pavement, allows the evaluation of variations in the structural signature using features extracted from periodograms, that is, from the estimation of the Power Spectral Density (PSD).

Specifically, the management of the acquired data is divided into two main phases: a pre-processing phase, where audio traces are divided into segments, and a subsequent processing phase, in which the extracted segments are analysed to extract features selected to characterize the structural condition of the road pavement.

**Figure 4:**
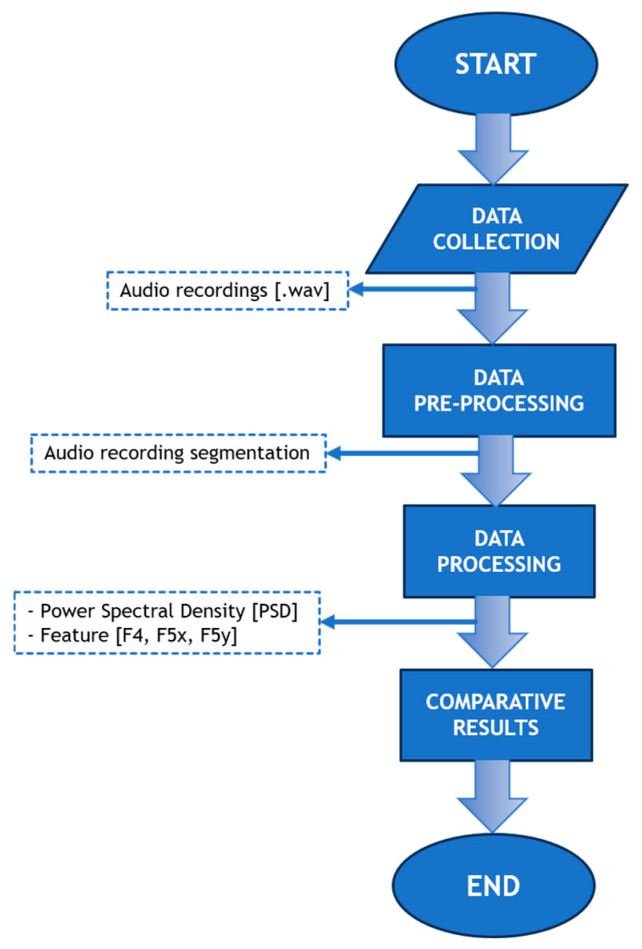
Flowchart of the data processing workflow.

Figure 4 provides a representation of the flowchart related to the data processing procedure.

### 3.1. Data Pre-Processing

**Figure 5:**
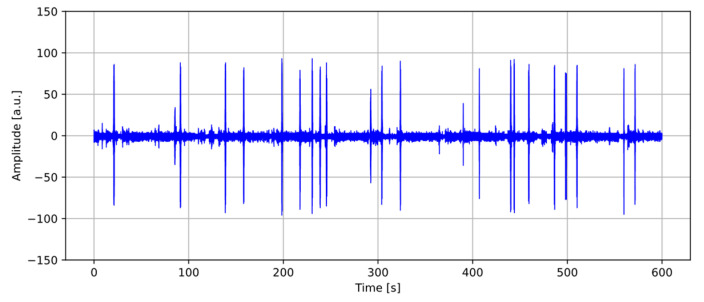
Waveform of the audio recording.

The pre-processing phase involves segmentation, which is the process by which an audio recording (Figure 5) is divided into segments containing significant portions of the signal. This enables targeted and detailed analysis of the relevant parts of the signal. This approach reduces computational load and allows for more efficient and accurate processing. Signal peaks are identified, isolated, and exported as .wav files, which are then available for further analysis.

The segmentation process involves resampling the acquired audio signals from 48,000 Hz to 1000 Hz using a Fourier Transform-based (FFT-based) approach implemented in Python through the SciPy library, in order to make the segmentation procedure lighter and more efficient. The purpose of the resampling is to generate a simplified version of the signal, with a data volume equal to approximately 2.1% of the original and a temporal resolution of one sample per millisecond, which is suitable for segment-detection operations. This operation does not affect the features, which, as shown later, are not extracted from the resampled signal but from the original 48,000 Hz trace, restricting the analysis to the 0–2500 Hz band. In future developments, the plan is to resample directly at 5000 Hz, applying a low-pass anti-aliasing filter beforehand to remove components above the Nyquist frequency and to perform the entire analysis on the resampled trace alone, without relying on the original signal.

On the audio signal sampled at 1000 Hz, the Root Mean Square value (RMS) is calculated. This value represents a measure of average power in the time domain, providing an indication of the energy content of the signal over a time interval.

The RMS value is used as an index of the signal’s power content over a specific time interval, to be compared with predefined threshold values to identify significant segments in the signal’s time series. Specifically, thresholds are defined that exceed the average power by a set number of standard deviations, suggesting the presence of anomalous events compared to background noise and, therefore, signals of interest.

This calculation is handy useful for representing the overall energy of the audio signal without being affected by the direction of oscillations (positive or negative). The RMS value is obtained as the square root of the mean of the squares of the samples.

(1) $$RMS=\sqrt{\frac{1}{N}\sum_{i=1}^{N}x_{i}^{2}}$$

where $x_{i}$ represents the intensity level of the audio signal for each sample, and *N* is the number of samples.

The total number of samples depends on the duration of the audio recording and the sampling frequency. As an example, if the audio recordings have a duration of 10 min (i.e., 600 s) and are sampled at 1000 Hz, the total number of samples will be 600,000.

*N* (total samples) = 1000 × 600 = 600,000 (2)

This value serves as the reference value for the entire audio recording.

Subsequently, a moving standard deviation is calculated on the recording converted to a sampling frequency of 1000 Hz. For this calculation, a sliding window centred on each point of the audio signal is used, with a window size of 100 samples. The window size is configurable and can be adjusted as needed. A larger window would include more points in the calculation, smoothing out local variations in the signal. This would result in a more uniform trend but would be less sensitive to rapid fluctuations.

The moving standard deviation is expressed as follows:

(3) $$\sigma =\sqrt{\frac{1}{N}\sum_{i=1}^{N}{\left(x_{i\;}-\bar{x}\right)}^{2}}$$

where *N* is the number of samples in the window, $x_{i\;}$ are the sample values within the window, and $\bar{x}$ is the mean of the values in the window.

It should be noted that in the case of a signal with a continuous component equal to zero, the values of *σ* and RMS coincide.

The process is iterated for each sample of the signal, with the window advancing one sample at a time, thereby generating a sequence of standard deviation values that encapsulate the local variability of the signal. The moving standard deviation is especially advantageous as it facilitates the detection of abrupt changes in the signal level, which may indicate noise or signals of interest, such as vehicular passages, and enables the identification of segments of the signal characterized by low variability, including periods of silence.

Although the moving standard deviation constitutes a smoothed representation of the original signal, it may still encapsulate undesired high-frequency components, specifically rapid oscillations that may compromise the integrity of the analysis. Consequently, a Butterworth low-pass filter is employed on the moving standard deviation to attenuate high-frequency components exceeding a predetermined cutoff frequency, while maintaining the integrity of the lower-frequency components. The general equation for the nth-order Butterworth low-pass filter is as follows:

(4) $$H\left(\omega \right)=\frac{1}{\sqrt{1+{\left(\frac{\omega }{\omega_{c}}\right)}^{2n}}}$$

where $H\left(\omega \right)$ denotes the frequency response of the filter, ω represents the angular frequency of the signal, $\omega_{c}$ denotes the cutoff frequency, and n indicates the order of the filter.

The Butterworth filter employed is a fourth-order filter, a choice that ensures a smooth transition between the passband (retained frequencies) and the stop band (attenuated frequencies), thereby minimizing distortions in the frequency response. The application of this filter reduces rapid oscillations and enhances low-frequency variations, which are often associated with more significant changes in the audio signal.

**Figure 6:**
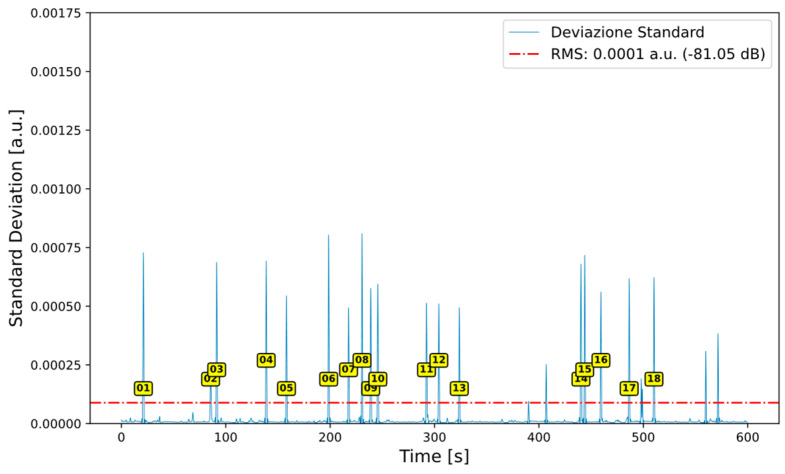
Identification of segmentation intervals.

After computing the Root Mean Square (RMS) value and applying the filtered moving standard deviation, intervals of interest are identified. These correspond to portions of the signal where the moving standard deviation exceeds the RMS threshold and are potentially associated with the passage of a specific vehicle category (Figure 6).

When the moving standard deviation exceeds the RMS value, the corresponding time is recorded as the beginning of a new interval. Conversely, when the standard deviation falls below the RMS value, the time is marked as the end of the interval. Intervals lasting more than one second are appended to the intervals_above_rms list for subsequent segment extraction, while shorter intervals are discarded.

The decision to exclude shorter intervals is based on preliminary testing, aimed at avoiding the inclusion of segments that may correspond to minor fluctuations or background noise rather than actual vehicular events. This temporal threshold thus contributes to more effectively isolating significant vehicle-related events and enhancing the robustness of the analysis.

At the end of the process, if the final interval remains open (i.e., extends to the end of the signal), it is still added to the intervals_above_rms list, if it satisfies the minimum duration requirement of one second.

Figure 6 highlights, through yellow markers, the time intervals selected based on a minimum duration criterion of 1 s.

Once these intervals are identified, the corresponding segments are extracted from the original audio signal, which is sampled at 48,000 Hz. Each segment is assigned a unique sequential identifier (ID), and the associated start and end times initially expressed in seconds are converted into sample indices by multiplying them by the sampling frequency (Table 1).

The resulting audio segments are then saved in .wav format and stored in a dedicated output directory for further analysis.

### 3.2. Data Processing

Once the various audio segments are extracted, the analysis proceeds by calculating their Power Spectral Density (PSD). This analysis enables the extraction of features (Table 2), in reference to previous studies related to this research and the waveform characteristics of vibro-acoustic signatures [24,25,28].

More specifically, the evaluation parameters for the processing are as follows:

▪ F4: The slope (in dB/Hz^2^) of the linear regression model fitted to the PSD curve within the 0–2500 Hz band. This parameter provides an estimate of the trend of energy decay across frequencies.
▪ F5x and F5y: A pair of features derived from the same PSD peak and therefore always extracted simultaneously. F5x (Hz) represents the dominant frequency (the abscissa of the maximum), while F5y (dB/Hz) represents the corresponding spectral amplitude (the ordinate of the maximum), within the 0–2500 Hz band.

**Figure 7:**
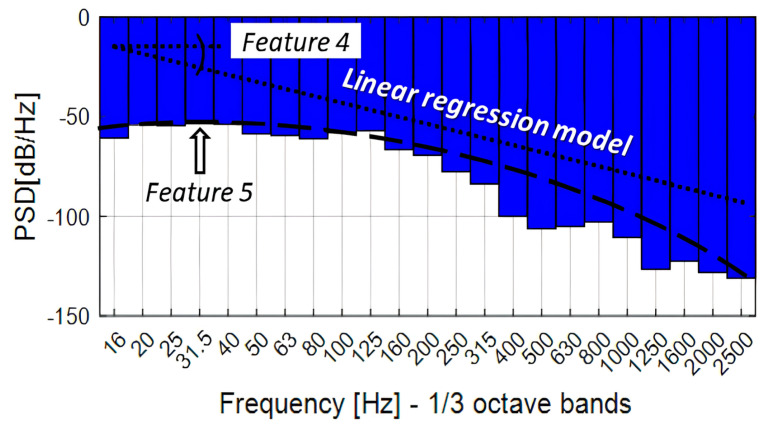
Feature F4 and F5 [25].

Figure 7 is a graphical representation of the features extracted in the frequency analysis domain. 

These parameters, based on the studies conducted, demonstrate a strong correlation with the values of the elastic modules [25], enabling the detection of variations in structural integrity with high probability, the identification of potential anomalies, and the monitoring of their evolution over time.

Variations in the elastic modulus affect both the propagation velocity and the attenuation of surface and body waves generated by the vehicle to pavement interaction. In particular, stiffer pavement layers tend to shift the dominant spectral components toward higher frequencies and reduce the low-frequency energy content, whereas damaged structures are expected to exhibit amplified low-frequency responses and a steeper spectral decay. The F4 and F5 parameters, therefore, act as indirect indicators of stiffness degradation, in accordance with previous vibro-acoustic and wave-based studies on road pavements [25,26,28].

The PSD is estimated using the Welch method. This method divides the segment extracted in the initial phase into overlapping parts, applies the FFT to each part, and finally averages the results.

Dividing the segment allows for the analysis of smaller intervals, reducing sensitivity to random fluctuations in the signal and providing more stable and accurate power estimates. The overlap between adjacent parts minimizes discontinuities that might occur at the boundaries of the segments.

In detail, for each part of the signal, the FFT is applied, which transforms the signal from the time domain to the frequency domain by decomposing it into its sinusoidal components at different frequencies. Subsequently, the magnitude of the FFT of each segment is squared to obtain the power spectrum, representing the distribution of the signal’s power as a function of frequency. Finally, the PSD estimate is obtained by averaging the power spectra of all segments, resulting in a more robust and reliable estimate compared to using a single FFT on the entire signal. This approach helps reduce variance and noise in the estimate.

Once the PSD is estimated, the analysis is limited to frequencies below 2500 Hz, focusing on the most relevant components of the signal and reducing the amount of data to process. This threshold was selected because most of the noise generated by vehicles, particularly from the contact between tyres and pavement, is concentrated in the low and mid-frequencies, typically below this value. Above 2500 Hz, power decreases, and the valuable information diminishes. Higher frequencies often contain less relevant information, as power tends to progressively decrease above this limit.

Subsequently, the features F5x and F5y are identified, corresponding to the maximum value of the PSD, and from the trend line, a linear representation of the power variation as a function of frequency can be obtained, allowing the extraction of the feature F4. 

**Figure 8:**
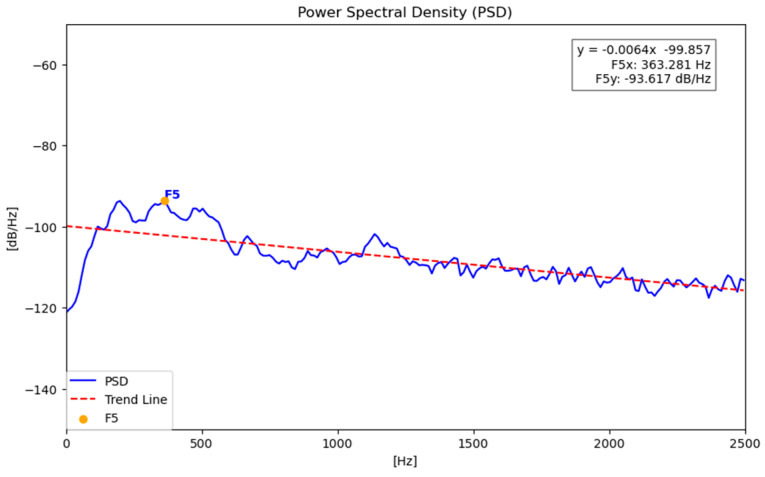
PSD example of an analysed segment.

Figure 8 illustrates the PSD and the extracted features. 

The variation in the features extracted from the signals is used as a simple and effective way to represent changes in the Structural Health Status (SHS) of the monitored roadway.

These features should allow for the characterization of the pavement’s structural condition and, at the same time, enable the correlation of specific variations in the recorded vibro-acoustic responses with the categories of passing vehicles.

The type of vehicle can be identified based on the frequency content of the recorded signals, as lower-frequency and longer-duration waves are typically associated with higher-class vehicles. Surface waves (e.g., Rayleigh waves) induced by heavy vehicles propagate at lower frequencies and with greater amplitude compared to those generated by lighter vehicles, allowing them to penetrate deeper into the pavement layers. In contrast, high-frequency components tend to attenuate rapidly with depth.

Structural damage is generally more sensitive to low-frequency excitation, which makes these components particularly effective for pavement condition monitoring and diagnostic purposes.

Based on these considerations, a field test campaign was conducted on a roadway open to regular traffic.

## 4. Road Test

The research campaign involved testing the prototype in a real road environment. Specifically, the campaign is still ongoing and began in July 2025 along the A4 highway on the Turin–Milan section (towards Turin), at km marker 107 + 300, where a prototype monitoring unit was installed with the support of SINA S.p.A. The choice of this location was influenced by the presence of an on-site WIM system, which enabled the assessment of measurement accuracy, as well as the identification of critical issues and potential future developments for the NDSPHM prototype system.

The highway section is characterized by a high volume of vehicular traffic, particularly during peak hours. As discussed later, the analyses presented in this study are based on a subset of the data collected during the experimental campaign. For the period considered, according to WIM data, the average daily traffic amounts to approximately 40,000 vehicles/day, consisting of 64% light vehicles and 36% heavy vehicles.

**Figure 9:**
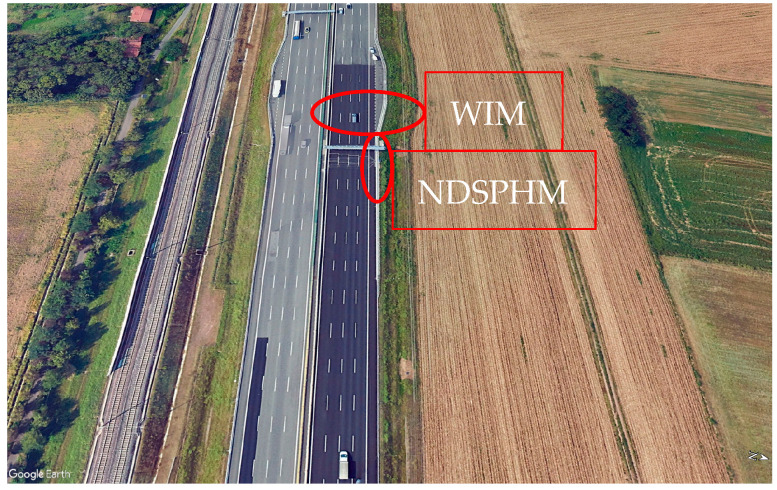
A4 Torino–Milano highway.

A satellite image of the installation area of the unit is shown in Figure 9.

The highway section where the prototype unit is installed consists of two separate carriageways, each with four lanes in each direction and an emergency lane.

**Figure 10:**
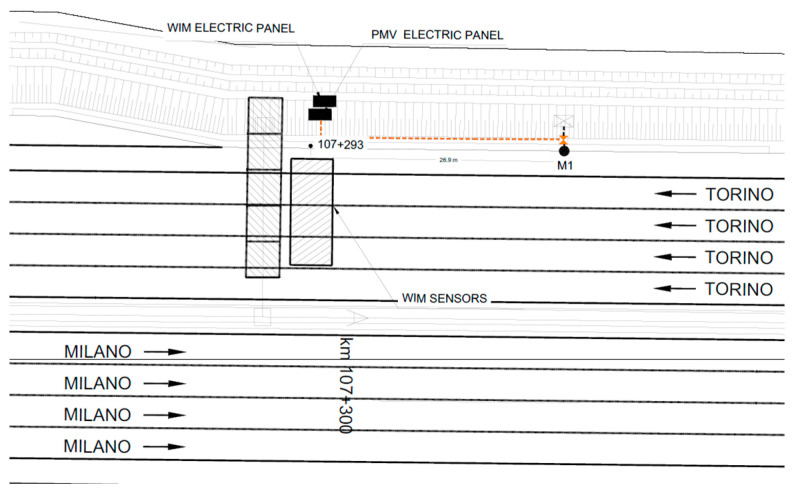
Installation plan on the A4 Torino–Milano highway.

**Figure 11:**
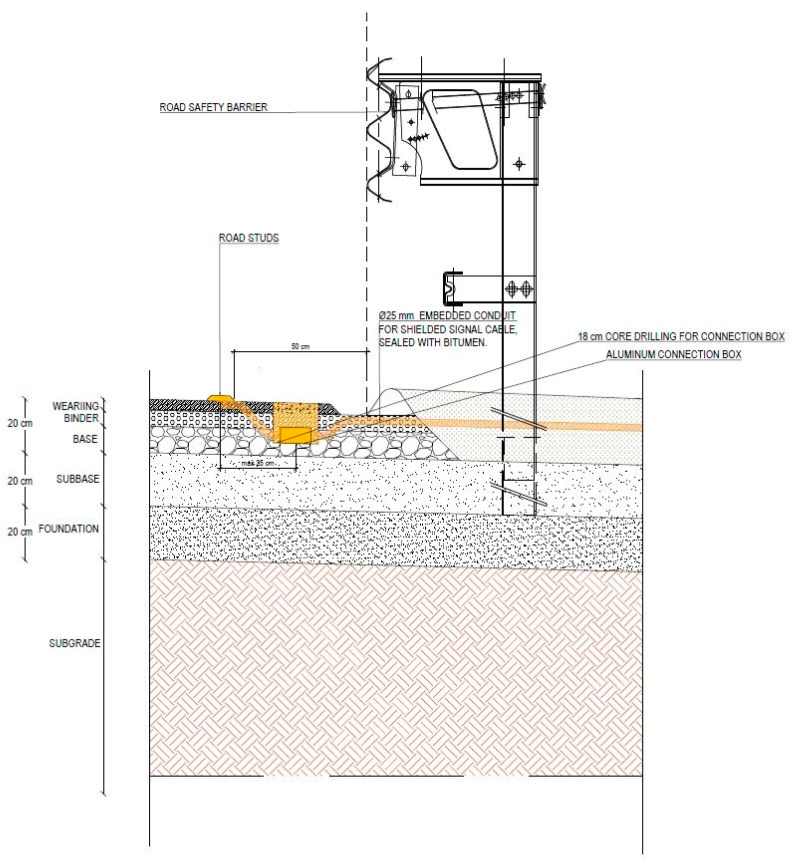
Detail of installation on the A4 Torino–Milano highway.

**Figure 12:**
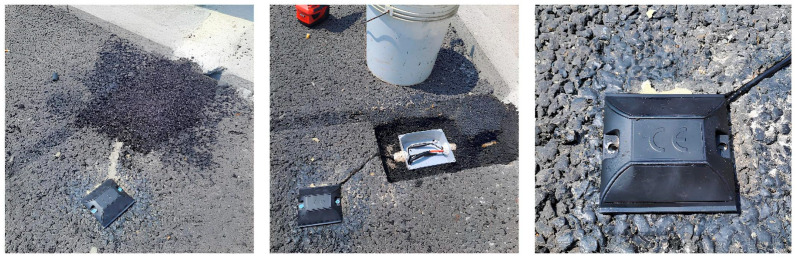
Sensor installation images.

As illustrated in the diagram in Figure 10, during the installation phase of the prototype and according to the configuration described in Section 2 of this paper, a local unit equipped with vibro-acoustic sensors and a temperature sensor was installed near the roadside safety barrier, in the direction of Torino, and connected to its respective central unit located outside the carriageway (Figure 11). Specifically, the local unit (M1) was installed 0.5 m from the safety barrier and 26.9 m from the WIM system (Figure 12).

As discussed later in the paper, the analysis of the data provided by the WIM system proved to be particularly useful for validating and interpreting the data collected by the sensors.

**Figure 13:**
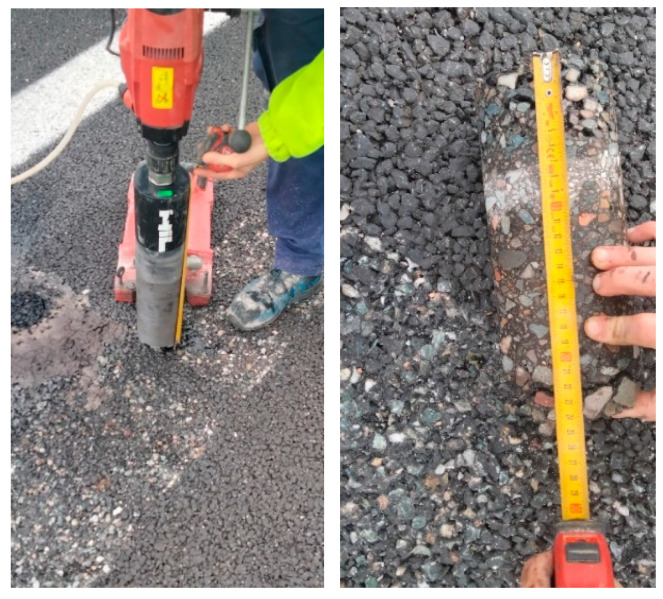
Core sampling.

To characterize the road section in terms of its pavement structure, a core sampling investigation was carried out (Figure 13). This procedure yielded detailed information regarding the composition and stratigraphy of the pavement layers, thereby validating the design specifications previously established for the same road segment.

The understanding of the pavement layers and their material composition enabled the representation of the pavement system using a multilayer linear elastic model.

Subsequently, for each sensor, three measurement positions were defined: one 1 m before, one 1 m after, and one in line with the sensor, all positioned transversely at 1 m from the sensor. Deflection data were acquired at each position using an HWD to support the structural evaluation.

**Figure 14:**
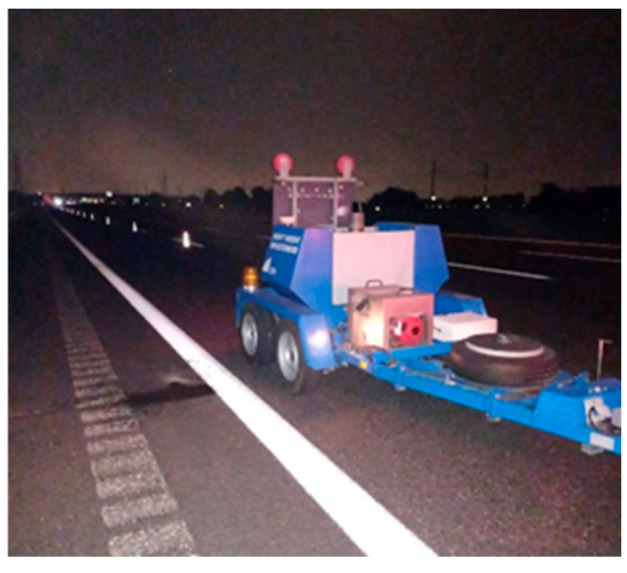
HWD measurements.

Figure 14 illustrates the instrumentation employed for the structural evaluation.

The collected data were analysed using ELMOD Version 6, a software tool designed to compute the material moduli within a pavement structure modelled as a multilayered, homogeneous, and isotropic elastic system over a semi-infinite subgrade.

## 5. Results

The processing results refer to a dataset of 170 audio recordings, encompassing both peak and off-peak traffic hours. Specifically, recordings from the first week of August 2025 were selected, following several preliminary tests aimed at stabilizing the acquisition system.

By applying the previously developed and described analysis procedure, individual segments were extracted, and a total of 7790 PSDs were computed, from which the results presented in the following sections are derived.

**Figure 15:**
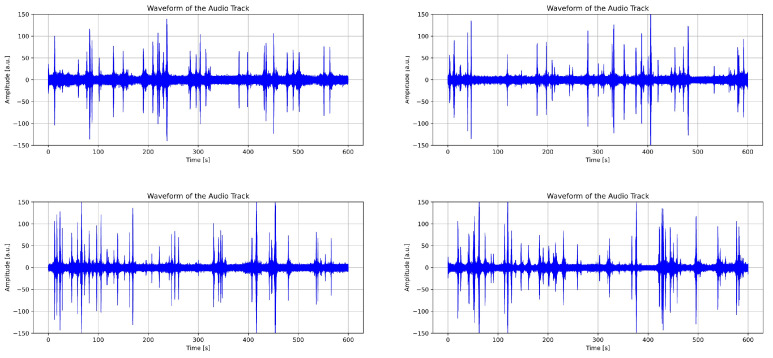
Waveforms of the audio recordings.

For demonstration purposes, selected examples of the waveforms of the analysed audio signals are presented in Figure 15.

**Figure 16:**
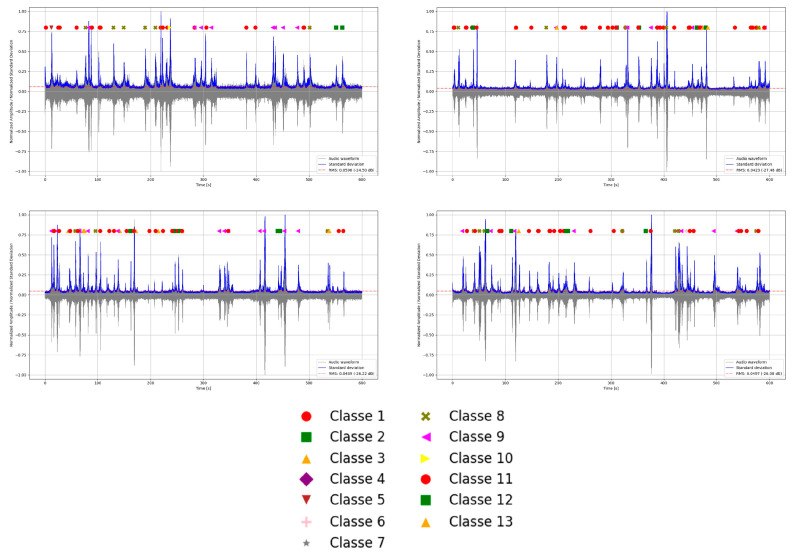
Vehicles associated with the signals.

Moreover, by leveraging the traffic data acquired from the WIM system during the recording period, it was possible to associate each analysis time interval with the corresponding information on vehicle classification, lane of travel, and operating speed, as presented in Figure 16.

Table 3 presents the results obtained from the analysed data and the elastic moduli calculated from HWD tests, referring to the same period as the audio acquisitions, during which the average pavement surface temperature was 31 °C.

Although the results are preliminary and based on a sample from a single pavement over a relatively limited period, they suggest promising outcomes, indicating data stability and significance, particularly for the F5 features, which have already shown positive correlation with the pavement’s elastic modulus in previous studies [25].

Regarding sensor installation, the road stud configuration has proven to be a practical solution, ensuring both high-quality acquisition of vibro-acoustic signal and road safety.

Specifically, although the detected intensity peaks are correlated with vehicle passages, there remains a risk that the captured vibro-acoustic waves may be influenced by environmental noise. However, this background noise can be effectively managed through the application of denoising filters directly to the signal.

## 6. Conclusions

This article presents an innovative system for vibro-acoustic monitoring of road pavements, based on microphones installed near the roadway to capture acoustic signatures generated by passing vehicles. Processing of the recorded signals enables the extraction of specific features that are useful for evaluating and monitoring the structural condition of pavement layers, with particular attention to the elastic modulus reduction and crack propagation.

Building on previous studies, a prototype has been developed to acquire vibro-acoustic responses induced by vehicular traffic, combined with a fully automated data processing framework for extracting the relevant parameters.

Experimental results have shown a high degree of stability and consistency in the mean and standard deviation values of the F5 parameters, which has proven to be the most promising feature to focus on in future investigations.

The data collection campaign is still ongoing and is generating a substantial amount of information. The initial field tests and the analyses conducted so far have provided significant indications regarding the sensitivity of the sensors’ acoustic features to dynamic loads and other environmental factors. Future work will focus on processing a larger dataset and on further investigating the correlation between the extracted features and traffic loads, in terms of both intensity and frequency. Additionally, it will be evaluated whether variations due to extreme weather conditions may affect the extracted parameters.

The system, which is still under development in both its hardware and software components, is intended as an alternative to conventional structural monitoring tools. Its goal is to enable the continuous analysis of large volumes of data, thereby supporting the development of predictive models for pavement deterioration.

Data acquisition activities will be extended to pavements with similar design characteristics but different levels of remaining service life, to build a comparative framework for the development of real-time pavement condition monitoring models, representing the ultimate objective of this experimental research.

Finally, the system is designed to operate, using multiple sensors distributed across the road network, enabling a comprehensive representation of pavement conditions managed by infrastructure authorities. Integration with Geographic Information Systems (GIS) allows for real-time, map-based visualization of updated pavement status. Additionally, the collected data are compliant with the formats required by Building Information Modeling (BIM) technologies, contributing to creating a digital twin of the infrastructure and supporting advanced asset management strategies.

## 7. Patents

A patent request number V3I240001306, already under process, could be referred to part of the work reported in this manuscript.

## Figures and Tables

**Table 1:** Segments extracted with a unique ID.

ID	Start Time (s)	End Time (s)
P001	20.408	21.742
P002	84.945	86.039
P003	90.667	91.953
P004	138.145	139.381
P005	157.565	158.743
P006	197.942	199.201
P007	217.049	218.245
P008	229.973	231.11
P009	238.225	239.55
P010	244.998	246.351
P011	291.703	292.898
P012	303.531	304.753
P013	323.146	324.313
P014	439.623	440.908
P015	443.322	444.497
P016	458.792	460.002
P017	485.871	487.067
P018	509.618	510.852

**Table 2:** Features extracted from the PSD.

Feature	F4	F5x	F5y
**Unit**	dB/Hz^2^	Hz	dB/Hz
**Physical** **Interpretation**	Indicator of the spectral decay trend within the analysed band	Dominant frequency of the vibro-acoustic response	Amplitude of the dominant peak

**Table 3:** Processing Results.

	F4 (dB/Hz^2^)	F5x (Hz)	F5y (dB/Hz)	E1 (MPa)	E2 (MPa)	E3 (MPa)
**Media**	−0.007	374.048	−87.599			
**Std. Dev.**	0.002	117.549	4.401			
**Max**	−0.0002	2285.156	−72.573			
**Min**	−0.015	70.313	−100.823			
				1637	2731	886

## Data Availability

The data presented in this study are available on request from the corresponding author due to legal reasons.

## Abbreviations

The following abbreviations are used in this manuscript:

PMS	Pavement Management System
FWD	Falling Weight Deflectometer
LWD	Light Weight Deflectometer
HWD	Heavy Weight Deflectometer
GPR	Ground Penetrating Radar
PSD	Power Spectral Density
RMS	Root Mean Square
FFT	Fast Fourier Transform
GIS	Geographic Information Systems
BIM	Building Information Modeling
